# Fluctuating renal function and the risk of incident atrial fibrillation: a nationwide population-based study

**DOI:** 10.1038/s41598-019-54528-w

**Published:** 2019-12-02

**Authors:** Soonil Kwon, So-Ryoung Lee, Eue-Keun Choi, Kyung-Do Han, Seokhun Yang, Seo-Young Lee, Hyun-Jung Lee, Inki Moon, Euijae Lee, Myung-Jin Cha, Woo-Hyun Lim, Seil Oh, Gregory Y. H. Lip

**Affiliations:** 1 Department of Internal Medicine, Seoul National University Hospital, Seoul, Republic of Korea; 2 Department of Medical Statistics, College of Medicine, The Catholic University of Korea, Seoul, Republic of Korea; 3 Department of Internal Medicine, Seoul Metropolitan Government-Seoul National University Boramae Medical Centre, Seoul, Republic of Korea; 4 Liverpool Centre for Cardiovascular Science, University of Liverpool and Liverpool Chest & Heart Hospital, Liverpool, United Kingdom; 5 Aalborg Thrombosis Research Unit, Department of Clinical Medicine, Aalborg University, Aalborg, Denmark

**Keywords:** Atrial fibrillation, Arrhythmias

## Abstract

Although chronic kidney disease is known to increase the risk of atrial fibrillation (AF), the impact of the variability of renal function on the risk of incident AF is unknown. We aimed to evaluate the association between variability of renal function and the risk of developing AF among the general population. We evaluated a total of 3,551,249 adults who had three annual health check-ups provided by the National Health Insurance Service. The variability of renal function was defined as GFR-VIM, which is variability independent of the mean (VIM) of creatinine-based estimated glomerular filtration rate (eGFR). The study population was divided into four groups (Q1-4) based on the quartiles of GFR-VIM, and the risks of incident AF by each group were compared. During a mean of 3.2 ± 0.5 years follow-up, incident AF occurred in 15,008 (0.42%) subjects. The incidence rates of AF increased from Q1 to Q4 (0.98, 1.42, 1.27, and 1.63 per 1,000 person-years, respectively). Adjusting with multiple variables, Q4 showed an increased risk of incident AF compared to Q1 (hazard ratio (HR) 1.125, 95% confidence interval (CI) 1.071–1.181). Variability of serum creatinine or other definitions of variability showed consistent results. On subgroup analyses, Q4 in males or those with a decreasing trend of eGFR had significantly increased risks of incident AF compared to Q1 (HR 1.127, 95% CI 1.082–1.175; and HR 1.115, 95% CI 1.059–1.173, respectively). High variability of eGFR was associated with an increased risk of incident AF, particularly in males or those with decreasing trends of eGFR during follow-up.

## Introduction

The prevalence of atrial fibrillation (AF) is increasing and is most frequently confronted arrhythmia in clinical practice^1–3^. Considering the impact of thromboembolism and mortality in patients with AF, long-term outcomes might be improved by suspecting silent AF, documenting the disease, and initiating appropriate management among a high-risk population^4–6^. Many risk factors are known to contribute to AF development^7^, but it is also essential to uncover and manage modifiable risk factors for incident AF^8^.

AF and renal functions are intimately interlinked with each other^9–14^. Multiple prospective studies have found that individuals with chronic kidney disease (CKD) have a higher risk of incident AF^10,12–14^. Other studies evaluated that not only the presence of CKD but also estimated glomerular filtration rate (eGFR) were associated with the risk of developing AF^9,10^. Conversely, AF also increases the risk of renal dysfunction^11,14^. However, one caveat is that renal function in most prior studies was ‘one-off’ assessments at baseline, often inferred by measuring (single) serum creatinine or cystatin c levels, despite the known visit-to-visit variability^15,16^. This renders an interpretation of the risk of AF to be difficult, especially among the individuals with high variability of renal function.

High variability of eGFR is known to increase the risk of progression or mortality of CKD^17,18^. However, there is a paucity of information on the association between variability of renal function and the risk of developing AF. Therefore, in this study, we investigated the impact of visit-to-visit variability of eGFR on the risk of incident AF among the general population using a nationwide population-based cohort.

## Results

During a mean of 3.2 ± 0.5 years of follow-up, AF occurred in 15,008 (0.42%) patients (incident rate = 1.33 per 1,000 person-years). The mean age of the total population was 44.2 years, and 68.5% were males. Characteristics of the study population categorized by the quartiles of variation independent of the mean of eGFR (GFR-VIM) are presented in Table 1. Compared to the individuals with the lowest eGFR variability (Q1), those with the highest eGFR variability (Q4) were older (mean age 46.9 vs. 40.8 years), less often male (57.3 vs. 83.9%), less smokers (current smoker 24.8 vs. 39.1%), less drinking (heavy drinking 6.8 vs. 10.2%), and having lower incomes (low income proportion 21.5 vs. 10.7%). All comorbidities were more prevalent in individuals with the highest eGFR variability (Q4).

**Table 1:** Baseline characteristics of the study population by the quartiles of GFR-VIM^*^.

	GFR-VIM				p-value
	Q1	Q2	Q3	Q4	
	N = 887,995	N = 887,624	N = 887,756	N = 887,874	
**Demographics**					
Age (year)	40.82 ± 10.33	46.22 ± 11.3	43.08 ± 11.19	46.86 ± 11.56	<0.0001
Age strata					<0.0001
20–39 (year)	438,073 (49.33)	250,892 (28.27)	364,998 (41.11)	247,700 (27.9)	
40–64 (year)	434,086 (48.88)	591,761 (66.67)	490,033 (55.2)	582,630 (65.62)	
≥65 (year)	15,836 (1.78)	44,971 (5.07)	32,725 (3.69)	57,544 (6.48)	
Male	745,029 (83.9)	470,308 (52.99)	709,076 (79.87)	508,415 (57.26)	<0.0001
Weight (kg)	69.86 ± 12.24	64.38 ± 11.91	69.08 ± 12.22	65.71 ± 12.28	<0.0001
Height (cm)	169.88 ± 7.9	164.75 ± 8.45	168.94 ± 7.97	165.2 ± 8.97	<0.0001
Waist circumference (cm)	81.87 ± 8.89	79.33 ± 9.42	81.77 ± 9.04	80.47 ± 9.27	<0.0001
Body mass index (kg/m^2^)	24.11 ± 3.31	23.61 ± 3.28	24.1 ± 3.3	23.96 ± 3.28	<0.0001
**Health habits**					
Smoking status					<0.0001
Never smoker	349,782 (39.39)	534,573 (60.23)	370,258 (41.71)	510,642 (57.51)	
Ex-smoker	191,405 (21.55)	160,314 (18.06)	200,448 (22.58)	156,987 (17.68)	
Current smoker	346,808 (39.06)	192,737 (21.71)	317,050 (35.71)	220,245 (24.81)	
Alcohol consumption					<0.0001
Never drinker	278,963 (31.41)	404,565 (45.58)	304,697 (34.32)	415,451 (46.79)	
Mild drinker	518,464 (58.39)	429,305 (48.37)	498,177 (56.12)	412,421 (46.45)	
Heavy drinker	90,568 (10.2)	53,754 (6.06)	84,882 (9.56)	60,002 (6.76)	
Regular exercise	205,801 (23.18)	208,041 (23.44)	213,473 (24.05)	211,217 (23.79)	<0.0001
Low income status	95,216 (10.72)	147,820 (16.65)	124,675 (14.04)	190,996 (21.51)	<0.0001
**Comorbidities**					
Chronic kidney disease	5,439 (0.61)	19,810 (2.23)	21,577 (2.43)	76,346 (8.6)	<0.0001
Diabetes	53,128 (5.98)	64,930 (7.32)	66,080 (7.44)	79,255 (8.93)	<0.0001
Hypertension	142,213 (16.02)	176,333 (19.87)	169,374 (19.08)	207,503 (23.37)	<0.0001
Dyslipidemia	137,710 (15.51)	168,229 (18.95)	156,547 (17.63)	197,614 (22.26)	<0.0001
**Laboratory results**^**†**^					
Serum creatinine (mg/dL)	0.92 ± 0.15	0.88 ± 0.18	0.93 ± 0.16	0.93 ± 0.31	<0.0001
eGFR (mL/min/1.73 m²)	90.67 ± 15.33	84.18 ± 16.28	89.13 ± 18.75	86.84 ± 24.26	<0.0001
Fasting glucose (mg/dL)	97.09 ± 20.5	97.48 ± 20.67	97.99 ± 21.75	98.48 ± 22.7	<0.0001
Systolic blood pressure (mmHg)	121.98 ± 12.85	120.35 ± 13.69	121.86 ± 13.15	121.31 ± 13.68	<0.0001
Diastolic blood pressure (mmHg)	76.83 ± 9.13	75.64 ± 9.46	76.68 ± 9.26	76.07 ± 9.45	<0.0001
Total cholesterol (mg/dL)	193.8 ± 35.06	194.34 ± 35.34	193.76 ± 35.46	195.32 ± 36.56	<0.0001
HDL (mg/dL)	53.87 ± 14.31	56.4 ± 14.91	54.3 ± 14.19	55.85 ± 14.66	<0.0001
LDL (mg/dL)	113.69 ± 39	114.03 ± 36.75	113.23 ± 41	114.14 ± 40.82	<0.0001
Triglyceride^‡^ (mg/dL)	117.48 (117.34–117.63)	105.07 (104.94–105.2)	116.86 (116.71–117)	111.97 (111.83–112.11)	<0.0001

Abbreviation: eGFR = estimated glomerular filtration rate calculated by MDRD equation; GFR-VIM = variability independent of mean of eGFR; HDL = high-density lipoprotein; LDL = low-density lipoprotein; Q1-4 = the 1^st^ ~ 4^th^ quartiles of GFR-VIM.

*GFR-VIM was calculated by 100 x SD/mean^β^, where β is the coefficient derived from curve fitting of eGFRs.

^†^All the values except triglyceride are presented as mean ± SD.

^‡^Presented as geometric mean with interquartile range.

Compared to Q1, Q4 had more CKD (8.6 vs. 0.6%), hypertension (23.4 vs. 16.0%), and dyslipidemia (22.3 vs. 15.5%). While there was no difference in mean creatinine in both groups (0.92 ± 0.15 in Q1 vs. 0.93 ± 0.31 mg/dL in Q4), there was a substantial difference in the baseline eGFR (90.67 ± 15.33 in Q vs. 86.84 ± 24.26 mL/min/1.73 m² in Q4). For other laboratory findings, subjects with Q4 had higher blood pressure, fasting serum glucose, and cholesterol levels compared with Q1. All comparisons showed statistical significance (p-values < 0.0001) due to the large numbers of the study population.

### GFR Variability and the Risk of AF

**Figure 1:**
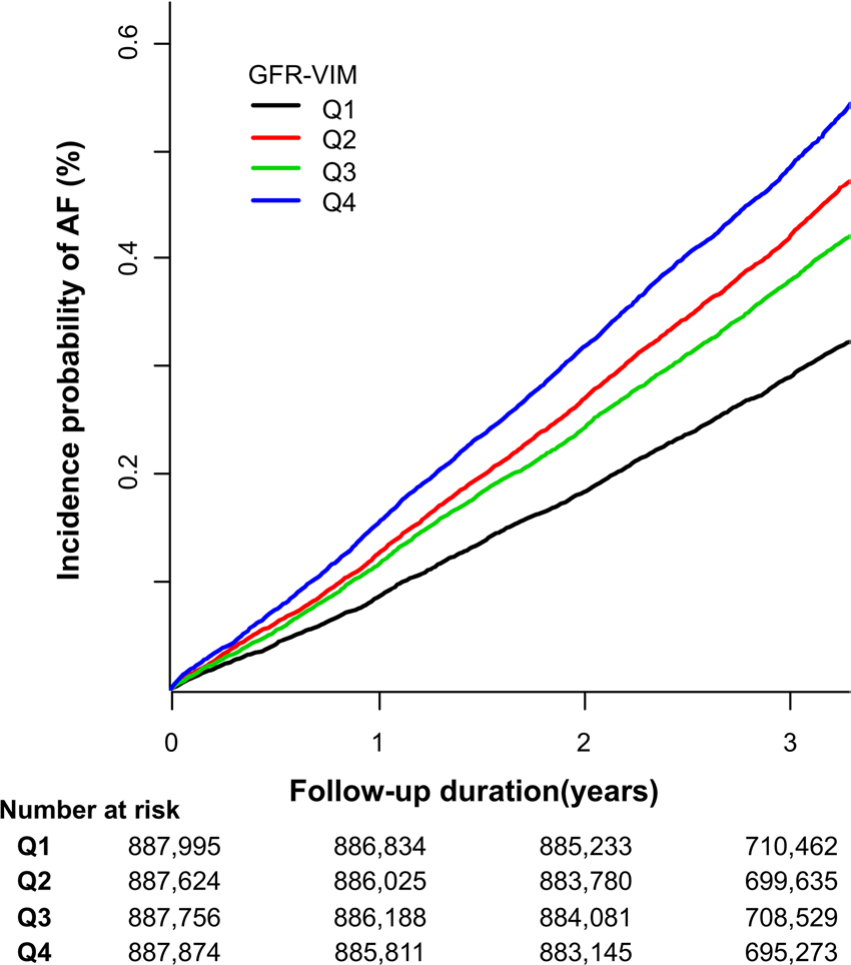
The cumulative incidences of incident atrial fibrillation by the quartiles of GFR-VIM. Compared to the individuals with the lowest variability of renal function (Q1), those with the highest variability of renal function (Q4) had higher crude incidence rates of atrial fibrillation. Abbreviation: AF = atrial fibrillation; eGFR = estimated glomerular filtration rate calculated by MDRD equation; GFR-VIM = variability independent of mean of eGFR.

**Figure 2:**
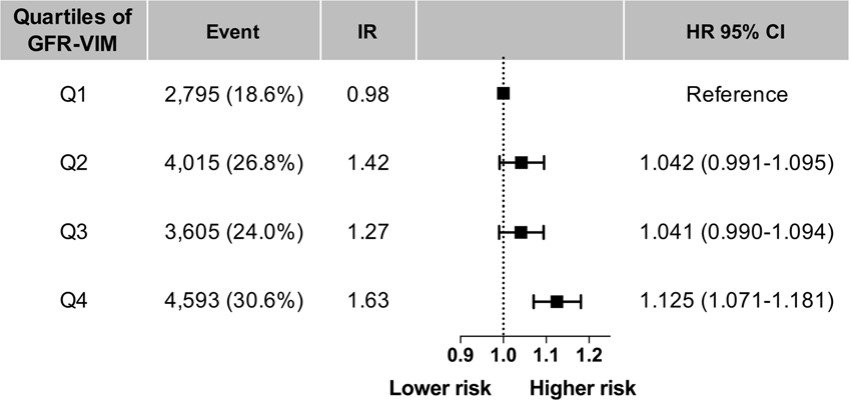
The risks of incident atrial fibrillation by the quartiles of GFR-VIM. With an adjusted model, the risk of incident AF was significantly increased in the group with the highest variability of renal function (Q4). For the individuals with a middle range of GFR-VIM (Q2 and Q3), there were no significant differences in AF risk compared to the group with the lowest variability of renal function (Q1). Abbreviation: CI = confidence interval; AF = atrial fibrillation; eGFR = estimated glomerular filtration rate calculated by MDRD equation; GFR-VIM = variability independent of mean of eGFR; HR = hazard ratio, adjusted by Model 2 (age, sex, smoking status, alcohol consumption, regular physical activity, low income level, body-mass index, hypertension, diabetes, and baseline eGFR); IR = incidence rate in 1,000 person-years.

The Kaplan-Meier survival curves and Cox regression analysis of incident AF by the quartiles of GFR-VIM are presented in Fig. 1. Compared to the individuals with the lowest quartile of GFR-VIM (Q1), those with the highest quartile of GFR-VIM (Q4) had higher crude incidence rates. Figure 2 and Supplementary Table S1 shows both incidence rates and risks of AF by quartiles of GFR-VIM. The incidence rate of AF increased from 0.98 to 1.63 per 1,000 person-years. In multivariate analysis adjusted for age, sex, smoking status, alcohol consumption, regular physical activity, low income level, body-mass index, hypertension, diabetes, and baseline eGFR, the risk of incident AF was also higher in the Q4 than Q1 (adjusted hazard ratio (HR) 1.125, 95% confidence interval (CI) 1.071–1.181, p-value < 0.001). For the individuals with a middle range of GFR-VIM (Q2 and Q3), there were no significant differences in AF risk compared to Q1 (adjusted HR 1.042, 95% CI 0.991–1.095 for Q2, adjusted HR 1.041, 95% CI 0.990–1.094 for Q3, all calculated by Model 2).

We analyzed the association between eGFR variability and incidence rate and the risk of AF by decile of eGFR (Supplementary Table S2 and Supplementary Fig. S1). There was a positive correlation between eGFR variability and the risk of developing AF. Compared to the individuals with the 1^st^ decile of GFR-VIM, those with higher than 7^th^ decile of GFR-VIM had a significantly higher risk of AF. Observing the trend of HRs by deciles of GFR-VIM, the individuals with extreme variability of eGFR (those with the 9^th^ and the last decile of GFR-VIM) were at the highest risk of incident AF (adjusted HR 1.176 and 1.202 respectively).

### Subgroup analyses

**Figure 3:**
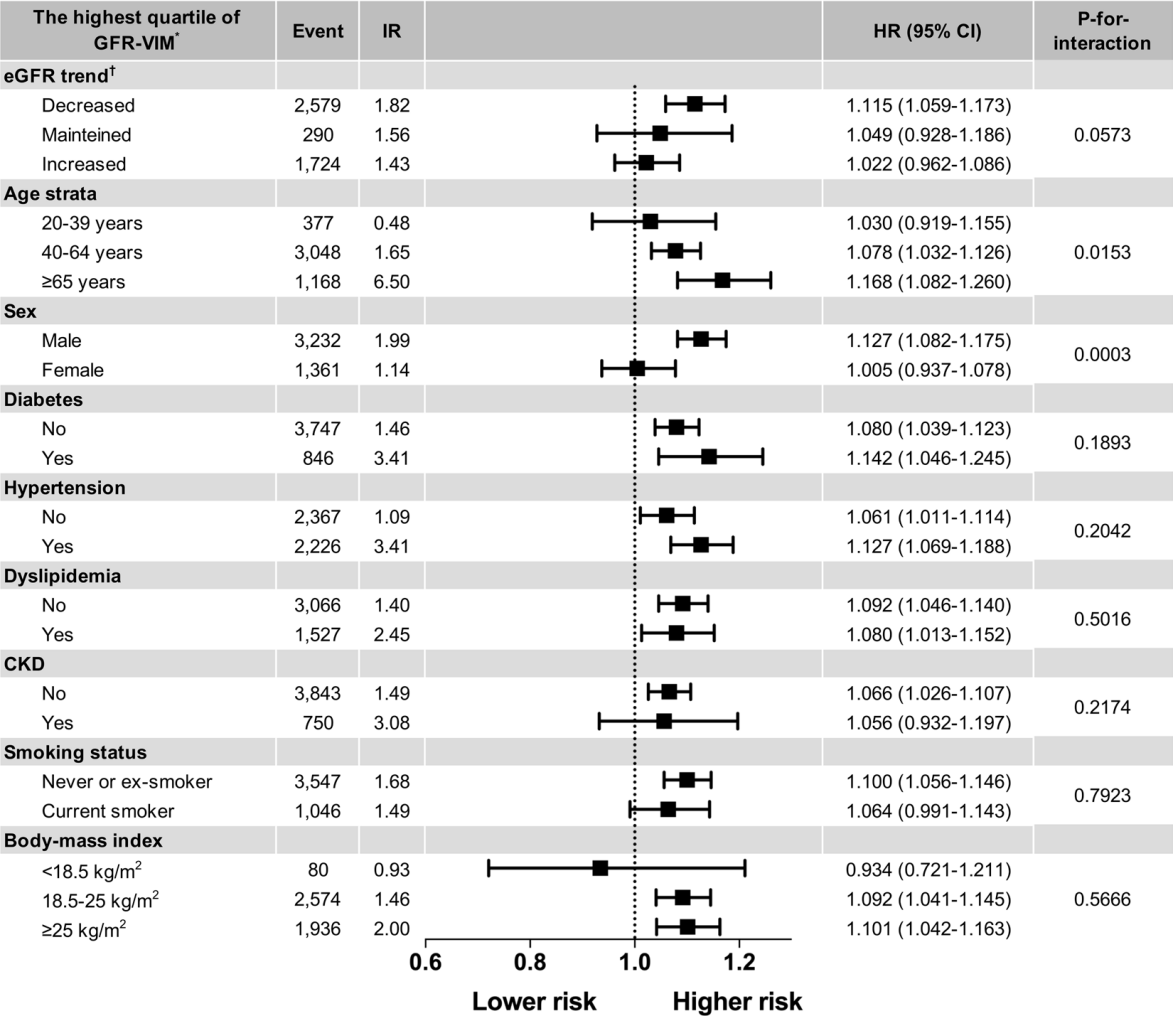
The risks of incident atrial fibrillation by various subgroups. Most subgroup analyses showed consistency with the main results, whereas age strata, sex, and eGFR trends showed significant interactions. For the subgroups of age strata, an increasing trend for developing AF risk was observed in older subjects. Males showed a higher risk of AF in those with higher eGFR variability. Among the subgroups by eGFR trends, only the subjects with a decreased eGFR trend had a significantly higher risk of AF in those with higher eGFR variability. Abbreviation: AF = atrial fibrillation; CI = confidence interval; CKD = chronic kidney disease; eGFR = estimated glomerular filtration rate calculated by MDRD equation; GFR-VIM = variability independent of mean of eGFR; HR = hazard ratio, adjusted by Model 2 (age, sex, smoking status, alcohol consumption, regular physical activity, low income level, body-mass index, hypertension, diabetes, and baseline eGFR); IR = incidence rate in 1,000 person-years. *Each subgroup represents the 4^th^ quartile of GFR-VIM with a suggested condition, whereas the corresponding lower three quartiles served as a reference. ^†^Individuals with decreased or increased eGFR trends were defined as the subgroups with the last eGFR lower or higher than 5% of the mean. Other cases were identified as maintained.

Most subgroup analyses showed consistency with the main results, whereas age strata, sex, and eGFR trends showed significant interactions (Fig. 3 and Supplementary Table S3). For the subgroups of age strata, an increasing trend for developing AF risk was observed in older subjects (adjusted HR 1.030, 95% CI 0.919–1.155 for 20–39 years; adjusted HR 1.078, 95% CI 1.032–1.126 for 40–64 years; and adjusted HR 1.168, 95% CI 1.082–1.260 for ≥65 years, where Q1–3 of each age stratum served as a reference, p-for-interaction = 0.0153). Males showed a higher risk of AF in those with higher eGFR variability (adjusted HR 1.127, 95% CI 1.082–1.175 for male Q4, where male Q1-3 served as a reference, p-for-interaction = 0.0003), whereas female did not. Among the subgroups by eGFR trends, subjects with a decreased eGFR trend had a significantly higher risk of AF in those with the highest eGFR variability (Q4), whereas those with maintained eGFR or increased eGFR trends showed a trend for increased risk of AF in those with the highest eGFR variability (Q4).

### Sensitivity analyses

To observe the consistent association between eGFR variability and AF risk, we calculated variability by standard deviation (SD), coefficient of variation (CV), and average real variability (ARV) (Supplementary Table S4 and Supplementary Fig. S2). The eGFR variability of the three methods was categorized into quartiles and calculated the risk of AF. In line with the variability independent of the mean (VIM) method, individuals with the highest variability had a significantly higher chance of AF than those with the lowest variability (adjusted HR 1.102, 95% CI 1.051–1.157 for SD; adjusted HR 1.137, 95% CI 1.085–1.191 for CV; and adjusted HR 1.100, 95% CI 1.048–1.153 for ARV, all in Model 2). Also, the variability of serum creatinine was calculated to remove the possible bias of age and sex (Supplementary Table S5 and Supplementary Fig. S3). Creatinine variability showed consistent results with increased risk of AF in the highest variability (adjusted HR 1.136, 95% CI 1.084–1.189 for SD, adjusted HR 1.133, 95% CI 1.081–1.187 for CV, adjusted HR 1.108, 95% CI 1.054–1.164 for VIM; and adjusted HR 1.106, 95% CI 1.060–1.155 for ARV, all in Model 2). The subjects with the highest variability showed a consistent increased risk of AF independent of the methods of variability, statistical models, or renal function measurements.

To adjust with the baseline renal function, we analyzed the association of the eGFR variability and the risk of AF according to baseline eGFR (eGFR ≥90, 60-89, <60 mL/min/1.73 m², Supplementary Table S6 and Supplementary Fig. S4). The number of events was 4,313 (28.74%), 9,507 (63.35%), and 1,188 (7.92%) in each subgroup respectively. After adjusted with other variables, individuals with the highest GFR-VIM showed an increased risk of AF only in those with eGFR 60–89 mL/min/1.73 m² (adjusted HR 1.106, 95% CI 1.039–1.177).

To remove the potential causality between AF and GFR variability, we censored incident AF that occurred within the first year of follow-up (Supplementary Table S7). In this sensitivity analysis, an additional 8.4% of AF risk was observed in those with the highest GFR-VIM (adjusted HR 1.084, 95% CI 1.024–1.149 for Q4). Although this risk was less than the result without censoring (HR 1.125, 95% CI 1.071–1.181), patients with the highest renal variability consistently showed a higher risk of AF.

## Discussion

To the best of our knowledge, this is the first report on evaluating the impact of the variability of renal functions on the risk of AF. The main findings of our study are as follows: (1) higher variability of eGFR was associated with an increased risk of incident AF, which was consistent regardless of methods of variability and metrics of renal function; and (2) the impact of variability of eGFR on the risk of AF only existed in males, age ≥40 years, or those with a decreasing eGFR trend.

Several studies have shown that impaired renal function was a risk factor of AF^10,12–14^. Watanabe *et al*. found subject with cystatin C-based eGFR <30 mL/min/1.73 m² had a 32% increased risk of AF compared to those with eGFR 30–59 mL/min/1.73 m² ^14^. Another prospective study showed that women had a significantly higher risk of AF (HR 1.36, 95% CI 1.00–1.84) only when there was severe impairment of renal function (cystatin C-based eGFR < 60 mL/min/1.73 m²)^19^. Also, Horio *et al*. demonstrated CKD was a powerful predictor of new-onset AF (HR 2.18), independent of left ventricular hypertrophy or left atrial dilatation^12^. According to a previous meta-analysis, eGFR < 60 mL/min/1.73 m² was the cut-off value which was associated with an increased risk of incident AF^10^. Across five categories of decreasing eGFR (≥90 (reference), 60–89, 45–59, 30–44, and <30 mL/min/1.73 m²), there was a stepwise increase in the risk of AF in the last three groups, but not in the group with eGFR 60–89 L/min/1.73 m² (HR 1.09, 95% CI 0.97–1.24)^10^.

Not only does the risk of AF increased according to renal dysfunction, but there is also evidence that new-onset AF increased the risk of renal dysfunction^11,14^. The presence of AF has been associated with the development of renal dysfunction (HR 1.77, 95% CI 1.50–2.10)^14^. The impact of AF on renal dysfunction was only observed in those with CKD^11^. Therefore, the association between AF and renal dysfunction seems to be bidirectional. This suggests that AF and CKD share many common pathophysiological processes. Of the latter, activation of the renin-angiotensin-aldosterone system (RAAS) can be considered first. The use of angiotensin-converting enzyme inhibitors or angiotensin receptor blockers in CKD patients has been reported to be effective in slowing the progression of CKD since RAAS is overtly activated in CKD^20^. Likewise, there are reports that RAAS also mediates AF development^21^, suggesting that exacerbation of RAAS may be associated with the progression of both diseases. Second, inflammation can be another mechanism in the progression of both diseases. In CKD, inflammatory indicators such as C-reactive protein and fibrinogen are elevated^22^, and this inflammatory milieu can affect the development of AF by mediating local inflammation in the left atrium^23^.

Recently, eGFR variability has attracted attention as a factor affecting renal function^17^. Fluctuating levels of serum creatinine, which changes nonlinearly like a saw-tooth, is known to have a detrimental effect on CKD progression and prognosis^24^. Unlike the decline in renal function, the impact of variability in renal function on AF is less known. Although the mechanism between the variability of renal function and AF is not well understood, other studies have reported that the variability of various metabolic indicators can be related to incident AF^25,26^. Based on previous reports, we would expect that variability in renal function would be associated with AF, provided that CKD and AF share some pathophysiological mechanisms. Also, we could hypothesize that high variability of renal function would be associated with AF development by promoting CKD and exacerbating RAAS modulation. Further large studies are needed to elucidate the associated mechanisms and causal associations.

There are several limitations to be noted. First, the cause of higher eGFR variability is unclear. Since our study used serum creatinine to calculate eGFR, any causes, such as fluctuating muscle mass, related to creatinine variability could influence eGFR variability. Second, a causal relationship between eGFR variability and incident AF needs more evidence given the observational design of our study. However, the sensitivity analysis to minimize the effects of reverse causality by excluding individuals with incident AF occurring within the first year showed consistent with the main results. Third, there was no data with cystatin C or combined creatinine-cystatin C-based eGFR, which is more accurate than the Modification of Diet in Renal Disease Study Group (MDRD) based eGFR^27^. This is an inherent limitation of the claims database of the National Health Insurance Service (NHIS) since it measures eGFR based on serum creatinine. Fourth, possible selection bias may originate from choosing the study population based on the number of health check-ups, since more regularly examined individuals may be prone to pay more attention to their health status. Also, there exists an inter-laboratory variation of serum creatinine measurement due to different calibration settings. Lastly, it would be difficult to confirm whether those with acute renal failure were excluded from the analysis. The health check-ups were performed in the outpatient clinic, so those currently being admitted to hospital for acute or chronic illness could not be enrolled. Therefore, we assumed that the cases of acute illness such as sepsis and other rapidly progressing acute kidney injury are extremely limited in this study.

In this nationwide population-based study, high eGFR variability was associated with an increased risk of incident AF. This was consistently observed across different methods of variability after adjusting potential confounders such as baseline eGFR. This association was particularly evident in males or those with decreasing trends of eGFR during follow-up.

## Methods

The NHIS of Korea was the source of the claims database used in the study. This claims database encompasses information of demography, prescription, procedures, and the diagnostic codes which are encoded in the International Classification of Disease, Tenth Revision, Clinical Modification. Since the NHIS is the single national insurer in Korea, it covers virtually all Koreans. Therefore, its database can represent comprehensive medical coverage of the entire Korean population. Since all the data are anonymized and can be accessed by researchers for public health studies, informed consent was exempted by the review board. The study protocol was approved by the Seoul National University Hospital Institutional Review Board (IRB No. 1811-132-987). All research was performed in accordance with relevant guidelines and regulations.

### Study design

**Figure 4:**
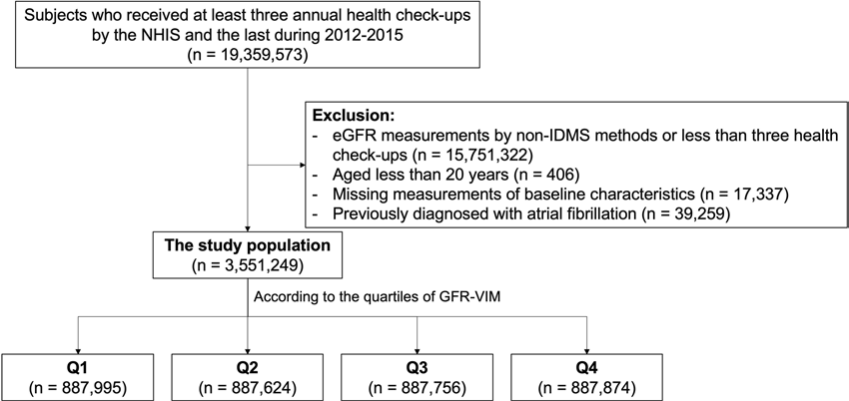
The flow chart of the study population. A total of 3,551,249 individuals were analyzed in the study. This population then categorized into the four groups by quartiles of GFR-VIM. Abbreviation: eGFR = estimated glomerular filtration rate calculated by MDRD equation; GFR-VIM = variability independent of mean of eGFR; IDMS = isotope dilution mass spectrometry; NHIS = national health insurance service.

The flow chart of the study population is presented in Fig. 4. For each who had health check-ups provided by the NHIS, the index date was set to be the date of the health check-up during 2014–2015. There was a total of 19,357,573 subjects who had a health check-up during 2014–2015. There were two categories of serum creatinine measurements during the health check-ups; an isotope dilution mass spectrometry (IDMS) method and a non-IDMS one. We excluded those who had less than three check-ups during 2012–2015 or those who measured serum creatinine by non-IDMS for the coherence of the analysis (excluding n = 15,751,322). Also, we excluded those aged less than twenty years (n = 406) or those with missing measurements of the variables (n = 17,337). Finally, we excluded those who had AF before the index date (n = 39,259). As a result, a total of 3,551,249 individuals were analyzed in our study. This population then categorized into four groups by quartiles of VIM^28^ of eGFR. From the lowest to the highest quartiles of GFR-VIM, the groups were named as Q1 to 4 accordingly.

### Definitions of measurements and variability

During the health check-up, every individual measured serum creatinine, and eGFR was calculated by a creatinine-based equation used from MDRD^29^. Other variables including blood pressure, cholesterol level, and fasting blood glucose were also measured. A standardized questionnaire by the NHIS evaluated individual health habits such as smoking status, alcohol consumption, and physical activities. Details of each question are listed in Supplementary Table S8. For comorbidities, we included hypertension, diabetes, dyslipidemia, and CKD, which were defined by operational definitions using diagnostic codes. Supplementary Table S9 summarizes the detailed definitions of the comorbidities used in the study. To define the individual variability of renal function, we used at least three eGFR values obtained from serial health check-ups. VIM was chosen to represent eGFR variability and was calculated as 100 x SD/mean^β^, where β is the coefficient derived from curve fitting using PROC NLIN procedure of the SAS package^28,30^.

### Study outcomes and follow-up

Incident AF, the primary outcome in this study, was defined as patients who had diagnostic codes of I48.0–48.4 or I48.9 during hospitalization or visiting the outpatient clinic more than 2 times. The index date was the date of the last health check-up during 2014–2015. The follow-up period was set from the index date to the time of incident AF or until December 31, 2018, whichever came first.

### Statistical analysis

For the Q1-4 quartiles of GFR-VIM, we used one-way analysis of variance and Pearson’s chi-square test to compare baseline characteristics. Incidence rates of AF were calculated as the number of events divided by 1,000 person-years during the follow-up. The survivals of the four groups were compared by the Kaplan-Meier analysis using log-rank tests. The risks of incident AF by the groups were evaluated by multivariate Cox proportional hazards regression models. The statistical model was adjusted for age, sex, smoking status, alcohol consumption, exercise habit, income level, body mass index (BMI) (Model 1), or further by the baseline eGFR and comorbidities including hypertension and diabetes (Model 2). We also re-categorized the study population in deciles of GFR-VIM to analyze trends between eGFR variability and incident AF risk. All the statistical analyses were performed in two-sided, and the statistical significance was defined as p-values of < 0.05. We performed statistical analyses with SAS 9.3 (SAS Institute Inc., Cary, North Carolina, USA).

### Subgroup analyses

Additional analyses of comparing the risks of AF between the Q4 versus Q1-3 (reference) were performed with the following subgroups: age strata (20–39, 40–64, and ≥65 years), sex, BMI (<18.5, 18.5–25, ≥25 kg/m^2^), comorbidities (CKD, hypertension, diabetes, and dyslipidemia), smoking status, and eGFR trends. The eGFR trends were grouped into three categories (decreased, maintained, and increased). Individuals with decreased or increased categories were those with the last eGFR lower or higher than 5% of the mean. The subgroup analyses were performed with multivariate Cox proportional hazards regression models with the same variables used in the main analysis. The statistical significance of the p-value between each group was defined as p-for-interaction of <0.1.

### Sensitivity analyses

To observe a consistent association between eGFR variability and incident AF risk, we calculated the variability by three other methods; SD, CV, and ARV^31^. Q1-4 categories were re-defined by the quartiles of each definition of variability, and multivariate Cox proportional hazards regression analyses were compared to each other. To remove the possible non-renal bias of eGFR, which incorporates variables including sex and age altogether, we calculated creatinine variability. Creatinine variability was calculated by VIM, SD, CV, and ARV. Cox proportional hazards regression analyses of accordingly categorized Q1-4 for each definition of creatinine variability were compared to each other. To observe whether the association between eGFR variability and incident AF risk was able to be reproduced in different classes of renal function, we performed additional sensitivity analysis. For the latter, the study population was divided into three groups by the baseline eGFR (<60, 60–89, and ≥90 mL/min/1.73 m²), and the Q1-4 for each group were re-defined by the quartiles of GFR-VIM of each class. Lastly, we performed another sensitivity analysis to remove the possible causal relationship between AF and eGFR variability. In this analysis, incident AF events that occurred within the first year were censored.

## Supplementary information

Supplementary materials

## Data Availability

The datasets generated and/or analyzed during the current study are not publicly available due to the national security policy on the health data of the NHIS.
