# Supplementary material for: Fluctuating renal function and the risk of incident atrial fibrillation: a nationwide population-based study
Source: Sci Rep. 2019 Dec 2;9:18055. doi: 10.1038/s41598-019-54528-w (PMC6889358; doi:10.1038/s41598-019-54528-w)
Supplement: Supplementary file 1 — Supplementary materials [file 41598_2019_54528_MOESM1_ESM.docx]

**Fluctuating renal function and the risk of incident atrial fibrillation: a nationwide population-based study**

Soonil Kwon, MD^a^, So-Ryoung Lee, MD^a^, Eue-Keun Choi, MD, PhD^a^, Kyung-Do Han, PhD^b^, Seokhun Yang, MD^a^, Seo-Young Lee, MD^a^, Hyun-Jung Lee, MD^a^, Inki Moon, MD^a^, Euijae Lee, MD^a^, Myung-Jin Cha, MD^a^, Woo-Hyun Lim, MD^c^, Seil Oh, MD, PhD, FHRS^a^, Gregory Y H Lip, MD^d,e^

^a^Department of Internal Medicine, Seoul National University Hospital, Seoul, Republic of Korea

^b^Department of Medical Statistics, College of Medicine, The Catholic University of Korea, Seoul, Republic of Korea

^c^Department of Internal Medicine, Seoul Metropolitan Government-Seoul National University Boramae Medical Centre, Seoul, Republic of Korea

^d^Liverpool Centre for Cardiovascular Science, University of Liverpool and Liverpool Chest & Heart Hospital, Liverpool, United Kingdom, and ^e^Aalborg Thrombosis Research Unit, Department of Clinical Medicine, Aalborg University, Aalborg, Denmark

**The first two authors contributed equally to this work**

**Short Title:** Variability of renal function and AF

**Corresponding Author**

Eue-Keun Choi, MD, PhD

Department of Internal Medicine, Seoul National University Hospital,

101, Daehak-ro, Jongno-gu, Seoul, 03080, Republic of Korea

Telephone: 82-2-2072-0688, Fax: 82-2-762-9662, E-mail: choiek17@snu.ac.kr

**Supplementary Materials**

- **Table S1**
- **Table S2**
- **Table S3**
- **Table S4**
- **Table S5**
- **Table S6**
- **Table S7**
- **Table S8**
- **Table S9**
- **Figure S1**
- **Figure S2**
- **Figure S3**
- **Figure S4**

**Table S1. The risk of incident AF by the variability of eGFR.**

|  | **Event** | **Follow-up duration (1,000 person-years)** | **Incidence rate (per 1,000 person-years)** | **Model 1** | **Model 2** |
| --- | --- | --- | --- | --- | --- |
| **GFR-VIM** |  |  |  |  |  |
| Q1 | 2,795 | 2,841,222 | 0.98 | 1 (ref.) | 1 (ref.) |
| Q2 | 4,015 | 2,832,119 | 1.42 | 1.050 (0.999-1.103) | 1.042 (0.991-1.095) |
| Q3 | 3,605 | 2,828,463 | 1.27 | 1.048 (0.997-1.101) | 1.041 (0.990-1.094) |
| Q4 | 4,593 | 2,815,963 | 1.63 | 1.151 (1.097-1.209) | 1.125 (1.071-1.181) |

Model 1: adjusted for age, sex, smoking status, alcohol consumption, regular physical activity, low-income level, and body-mass index.

Model 2: adjusted for age, sex, smoking status, alcohol consumption, regular physical activity, low-income level, body-mass index, hypertension, diabetes, and baseline eGFR.

Abbreviation: eGFR =estimated glomerular filtration rate calculated by MDRD equation; GFR-VIM =variability independent of the mean of eGFR; Q1-4 =the 1^st^ ~ 4^th^ quartiles of GFR-VIM.

**Table S2. The risk of incident atrial fibrillation by deciles of GFR-VIM.**

| **GFR-VIM** | **Event** | **Follow-up duration (1,000 person-years)** | **Incidence rate (per 1,000 person-years)** | **Model 1** | **Model 2** |
| --- | --- | --- | --- | --- | --- |
| D1 | 1,392 | 1,139,118 | 1.22 | 1 (ref.) | 1 (ref.) |
| D2 | 746 | 1,132,249 | 0.66 | 1.092 (0.996-1.197) | 1.094 (0.997-1.199) |
| D3 | 1,401 | 1,140,571 | 1.23 | 1.016 (0.942-1.095) | 1.019 (0.946-1.099) |
| D4 | 1,977 | 1,138,589 | 1.74 | 1.087 (1.014-1.165) | 1.080 (1.008-1.158) |
| D5 | 1,294 | 1,122,815 | 1.15 | 1.173 (1.083-1.271) | 1.160 (1.071-1.257) |
| D6 | 1,058 | 1,133,842 | 0.93 | 1.060 (0.978-1.149) | 1.061 (0.979-1.150) |
| D7 | 1,910 | 1,132,531 | 1.69 | 1.104 (1.030-1.184) | 1.094 (1.020-1.173) |
| D8 | 1,392 | 1,124,879 | 1.24 | 1.091 (1.013-1.176) | 1.082 (1.004-1.166) |
| D9 | 1,825 | 1,128,092 | 1.62 | 1.195 (1.114-1.282) | 1.176 (1.097-1.261) |
| D10 | 2,013 | 1,125,083 | 1.79 | 1.242 (1.160-1.331) | 1.202 (1.122-1.288) |

Model 1: adjusted for age, sex, smoking status, alcohol consumption, regular physical activity, low-income level, and body-mass index.

Model 2: adjusted for age, sex, smoking status, alcohol consumption, regular physical activity, low-income level, body-mass index, hypertension, diabetes, and baseline eGFR.

Abbreviation: eGFR =estimated glomerular filtration rate calculated by MDRD equation; GFR-VIM =variability independent of the mean of eGFR.

**Table S3. The various subgroup analyses**

|  | **Subgroup** |  | **Event** | **Duration** | **Incidence rate (per 1,000 person-years)** | **Model 2** | **p-for-interaction** |
| --- | --- | --- | --- | --- | --- | --- | --- |
| **eGFR trend**^*^ | Decreased | Q1-3 | 4,280 | 3,338,809 | 1.28 | 1 (ref.) | 0.0573 |
|  |  | Q4 | 2,579 | 1,420,673 | 1.82 | 1.115 (1.059-1.173) |  |
|  | Maintained | Q1-3 | 2,984 | 2,507,714 | 1.19 | 1 (ref.) |  |
|  |  | Q4 | 290 | 186,341 | 1.56 | 1.049 (0.928-1.186) |  |
|  | Increased | Q1-3 | 3,151 | 2,655,281 | 1.19 | 1 (ref.) |  |
|  |  | Q4 | 1,724 | 1,208,949 | 1.43 | 1.022 (0.962-1.086) |  |
| **Age strata** | 20-39 years | Q1-3 | 1,509 | 3,355,307 | 0.45 | 1 (ref.) | 0.0153 |
|  |  | Q4 | 377 | 786,826 | 0.48 | 1.030 (0.919-1.155) |  |
|  | 40-64 years | Q1-3 | 7,257 | 4,853,760 | 1.50 | 1 (ref.) |  |
|  |  | Q4 | 3,048 | 1,849,372 | 1.65 | 1.078 (1.032-1.126) |  |
|  | ≥65 years | Q1-3 | 1,649 | 292,738 | 5.63 | 1 (ref.) |  |
|  |  | Q4 | 1,168 | 179,765 | 6.50 | 1.168 (1.082-1.260) |  |
| **Sex** | Male | Q1-3 | 8,462 | 6,169,691 | 1.37 | 1 (ref.) | 0.0003 |
|  |  | Q4 | 3,232 | 1,621,475 | 1.99 | 1.127 (1.082-1.175) |  |
|  | Female | Q1-3 | 1,953 | 2,332,113 | 0.84 | 1 (ref.) |  |
|  |  | Q4 | 1,361 | 1,194,489 | 1.14 | 1.005 (0.937-1.078) |  |
| **Diabetes** | No | Q1-3 | 8,943 | 7,919,995 | 1.13 | 1 (ref.) | 0.1893 |
|  |  | Q4 | 3,747 | 2,567,589 | 1.46 | 1.080 (1.039-1.123) |  |
|  | Yes | Q1-3 | 1,472 | 581,810 | 2.53 | 1 (ref.) |  |
|  |  | Q4 | 846 | 248,374 | 3.41 | 1.142 (1.046-1.245) |  |
| **Hypertension** | No | Q1-3 | 6,266 | 6,954,512 | 0.90 | 1 (ref.) | 0.2042 |
|  |  | Q4 | 2,367 | 2,162,839 | 1.09 | 1.061 (1.011-1.114) |  |
|  | Yes | Q1-3 | 4,149 | 1,547,293 | 2.68 | 1 (ref.) |  |
|  |  | Q4 | 2,226 | 653,124 | 3.41 | 1.127 (1.069-1.188) |  |
| **Dyslipidaemia** | No | Q1-3 | 7,552 | 7,034,578 | 1.07 | 1 (ref.) | 0.5016 |
|  |  | Q4 | 3,066 | 2,193,489 | 1.40 | 1.092 (1.046-1.140) |  |
|  | Yes | Q1-3 | 2,863 | 1,467,227 | 1.95 | 1 (ref.) |  |
|  |  | Q4 | 1,527 | 622,475 | 2.45 | 1.080 (1.013-1.152) |  |
| **Chronic kidney disease** | No | Q1-3 | 9,977 | 8,350,978 | 1.19 | 1 (ref.) | 0.2174 |
|  |  | Q4 | 3,843 | 2,572,457 | 1.49 | 1.066 (1.026-1.107) |  |
|  | Yes | Q1-3 | 438 | 150,826 | 2.90 | 1 (ref.) |  |
|  |  | Q4 | 750 | 243,506 | 3.08 | 1.056 (0.932-1.197) |  |
| **Smoking status** | Never or ex- | Q1-3 | 7,353 | 5757,134 | 1.28 | 1 (ref.) | 0.7923 |
|  |  | Q4 | 3,547 | 2,114,476 | 1.68 | 1.100 (1.056-1.146) |  |
|  | Current | Q1-3 | 3,062 | 2,744,670 | 1.12 | 1 (ref.) |  |
|  |  | Q4 | 1,046 | 701,488 | 1.49 | 1.064 (0.991-1.143) |  |
| **Body-mass index** | <18.5 kg/m^2^ | Q1-3 | 231 | 283,960 | 0.81 | 1 (ref.) | 0.5666 |
|  |  | Q4 | 80 | 86,287 | 0.93 | 0.934 (0.721-1.211) |  |
|  | 18.5-25 kg/m^2^ | Q1-3 | 5,876 | 5,293,381 | 1.11 | 1 (ref.) |  |
|  |  | Q4 | 2,574 | 1,759,823 | 1.46 | 1.092 (1.041-1.145) |  |
|  | ≥25 kg/m^2^ | Q1-3 | 4,302 | 2,916,974 | 1.47 | 1 (ref.) |  |
|  |  | Q4 | 1,936 | 967,925 | 2.00 | 1.101 (1.042-1.163) |  |

Model 2: adjusted for age, sex, smoking status, alcohol consumption, regular physical activity, low-income level, body-mass index, hypertension, diabetes, and baseline eGFR.

Abbreviation: eGFR =estimated glomerular filtration rate calculated by MDRD equation.

*Individuals with decreased or increased eGFR trends were defined as the subgroups with the last eGFR lower or higher than 5% of the mean. Other cases were defined as maintained.

**Table S4. The risk of incident atrial fibrillation by the variability of serum creatinine.**

|  | **Event** | **Follow-up duration (1,000 person-years)** | **Incidence rate (per 1,000 person-years)** | **Model 1** | **Model 2** |
| --- | --- | --- | --- | --- | --- |
| **GFR-VIM** |  |  |  |  |  |
| Q1 | 2,795 | 2,841,222 | 0.98 | 1 (ref.) | 1 (ref.) |
| Q2 | 4,015 | 2,832,119 | 1.42 | 1.050 (0.999-1.103) | 1.042 (0.991-1.095) |
| Q3 | 3,605 | 2,828,463 | 1.27 | 1.048 (0.997-1.101) | 1.041 (0.990-1.094) |
| Q4 | 4,593 | 2,815,963 | 1.63 | 1.151 (1.097-1.209) | 1.125 (1.071-1.181) |
| **GFR-SD** |  |  |  |  |  |
| Q1 | 4,017 | 2,852,655 | 1.41 | 1 (ref.) | 1 (ref.) |
| Q2 | 3,572 | 2,837,936 | 1.26 | 0.984 (0.940-1.029) | 0.991 (0.947-1.037) |
| Q3 | 3,763 | 2,824,210 | 1.33 | 1.028 (0.983-1.075) | 1.043 (0.997-1.092) |
| Q4 | 3,656 | 2,802,967 | 1.30 | 1.074 (1.026-1.123) | 1.102 (1.051-1.157) |
| **GFR-CV** |  |  |  |  |  |
| Q1 | 3,613 | 2,853,505 | 1.27 | 1 (ref.) | 1 (ref.) |
| Q2 | 3,517 | 2,827,499 | 1.24 | 1.040 (0.992-1.090) | 1.050 (1.001-1.101) |
| Q3 | 3,786 | 2,829,505 | 1.34 | 1.034 (0.988-1.082) | 1.037 (0.990-1.085) |
| Q4 | 4,092 | 2,807,259 | 1.46 | 1.133 (1.083-1.186) | 1.137 (1.085-1.191) |
| **GFR-ARV** |  |  |  |  |  |
| Q1 | 3,869 | 2,851,647 | 1.36 | 1 (ref.) | 1 (ref.) |
| Q2 | 3,751 | 2,835,710 | 1.32 | 1.031 (0.986-1.079) | 1.034 (0.988-1.081) |
| Q3 | 3,756 | 2,828,681 | 1.33 | 1.043 (0.997-1.091) | 1.053 (1.006-1.102) |
| Q4 | 3,632 | 2,801,729 | 1.3 | 1.081 (1.033-1.132) | 1.100 (1.048-1.153) |

Model 1: adjusted for age, sex, smoking status, alcohol consumption, regular physical activity, low-income level, and body-mass index.

Model 2: adjusted for age, sex, smoking status, alcohol consumption, regular physical activity, low income level, body-mass index, hypertension, diabetes, and baseline eGFR.

Abbreviation: eGFR =estimated glomerular filtration rate calculated by MDRD equation; GFR-ARV =average real variability of eGFR; GFR-CV =coefficient of variation of eGFR; GFR-SD =standard deviation of eGFR; GFR-VIM =variability independent of mean of eGFR; Q1-4 =the 1^st^ ~ 4^th^ quartiles of each category.

**Table S5. The risk of incident atrial fibrillation by the variability of serum creatinine.**

|  | **Event** | **Follow-up duration (1,000 person-years)** | **Incidence rate (per 1,000 person-years)** | **Model 1** | **Model 2** |
| --- | --- | --- | --- | --- | --- |
| **Cr-VIM** |  |  |  |  |  |
| Q1 | 3,920 | 2,989,071 | 1.31 | 1(ref.) | 1(ref.) |
| Q2 | 3,730 | 2,735,801 | 1.36 | 1.004 (0.960-1.050) | 1.011 (0.967-1.058) |
| Q3 | 3,672 | 2,828,601 | 1.3 | 1.050 (1.003-1.099) | 1.070 (1.021-1.122) |
| Q4 | 3,686 | 2,764,294 | 1.33 | 1.080 (1.031-1.131) | 1.108 (1.054-1.164) |
| **Cr-SD** |  |  |  |  |  |
| Q1 | 3,110 | 2,645,717 | 1.18 | 1(ref.) | 1(ref.) |
| Q2 | 3,873 | 3,103,847 | 1.25 | 1.047 (0.999-1.098) | 1.045 (0.997-1.095) |
| Q3 | 3,523 | 2,702,660 | 1.3 | 1.049 (0.999-1.100) | 1.044 (0.995-1.095) |
| Q4 | 4,502 | 2,865,544 | 1.57 | 1.156 (1.104-1.210) | 1.136 (1.084-1.189) |
| **Cr-CV** |  |  |  |  |  |
| Q1 | 3,761 | 2,949,258 | 1.28 | 1(ref.) | 1(ref.) |
| Q2 | 3,244 | 2,636,480 | 1.23 | 1.030 (0.982-1.080) | 1.046 (0.996-1.098) |
| Q3 | 3,985 | 2,938,434 | 1.36 | 1.041 (0.995-1.088) | 1.046 (1.000-1.094) |
| Q4 | 4,018 | 2,793,596 | 1.44 | 1.125 (1.075-1.177) | 1.133 (1.081-1.187) |
| **Cr-ARV** |  |  |  |  |  |
| Q1 | 4,030 | 3,438,999 | 1.17 | 1(ref.) | 1(ref.) |
| Q2 | 3,704 | 2,893,740 | 1.28 | 1.039 (0.994-1.087) | 1.033 (0.988-1.080) |
| Q3 | 2,673 | 2,038,659 | 1.31 | 1.043 (0.993-1.095) | 1.036 (0.987-1.088) |
| Q4 | 4,601 | 2,946,369 | 1.56 | 1.129 (1.082-1.178) | 1.106 (1.060-1.155) |

Model 1: adjusted for age, sex, smoking status, alcohol consumption, regular physical activity, low-income level, and body-mass index.

Model 2: adjusted for age, sex, smoking status, alcohol consumption, regular physical activity, low-income level, body-mass index, hypertension, diabetes, and baseline eGFR.

Abbreviation: Cr-ARV =average real variability of serum creatinine; Cr-CV =coefficient of variation of serum creatinine; CR-SD =standard deviation of serum creatinine; Cr-VIM =variability independent of the mean of serum creatinine; eGFR =estimated glomerular filtration rate calculated by MDRD equation; Q1-4 =the 1st ~ 4th quartiles of each category.

**Table S6. The risk of incident atrial fibrillation by the variability of serum creatinine and baseline eGFR classes**^*^**.**

| **eGFR class** | **GFR-VIM** | **Event** | **Follow-up duration (1,000 person-years)** | **Incidence rate (per 1,000 person-years)** | **Model 1** | **Model 2** |
| --- | --- | --- | --- | --- | --- | --- |
| ≥90 mL/min/1.73 m² | Q1 | 838 | 1,077,765 | 0.78 | 1(ref.) | 1(ref.) |
|  | Q2 | 1,063 | 1,074,847 | 0.99 | 1.011 (0.921-1.110) | 1.016 (0.925-1.116) |
|  | Q3 | 1,090 | 1,076,671 | 1.01 | 1.023 (0.934-1.120) | 1.021 (0.932-1.118) |
|  | Q4 | 1,322 | 1,075,290 | 1.23 | 1.063 (0.972-1.164) | 1.044 (0.953-1.144) |
| 60-89 mL/min/1.73 m² | Q1 | 1,805 | 1,664,711 | 1.08 | 1(ref.) | 1(ref.) |
|  | Q2 | 2,672 | 1,660,100 | 1.61 | 1.075 (1.012-1.143) | 1.061 (0.998-1.128) |
|  | Q3 | 2,339 | 1,653,161 | 1.41 | 1.062 (0.998-1.130) | 1.044 (0.981-1.111) |
|  | Q4 | 2,691 | 1,640,892 | 1.64 | 1.142 (1.074-1.214) | 1.106 (1.039-1.177) |
| <60 mL/min/1.73 m² | Q1 | 257 | 99,196 | 2.59 | 1(ref.) | 1(ref.) |
|  | Q2 | 284 | 98,943 | 2.87 | 1.102 (0.931-1.305) | 1.093 (0.923-1.294) |
|  | Q3 | 290 | 98,359 | 2.95 | 1.149 (0.971-1.360) | 1.104 (0.933-1.306) |
|  | Q4 | 357 | 97,835 | 3.65 | 1.439 (1.225-1.690) | 1.106 (0.927-1.321) |

Model 1: adjusted for age, sex, smoking status, alcohol consumption, regular physical activity, low-income level, and body-mass index.

Model 2: adjusted for age, sex, smoking status, alcohol consumption, regular physical activity, low-income level, body-mass index, hypertension, diabetes, and baseline eGFR.

Abbreviation: eGFR =estimated glomerular filtration rate calculated by MDRD equation; GFR-VIM =variability independent of the mean of eGFR; Q1-4 =the 1st ~ 4th quartiles of each category.

*The study population was divided into three classes by the baseline eGFR (<60, 60-89, and ≥90 mL/min/1.73 m²), and the Group 1-4 for each class were re-defined by the quartiles of GFR-VIM of each class.

**Table S7. The risk of incident atrial fibrillation by the variability of eGFR, censoring events occurred within the first year during the follow-up.**

| **GFR-VIM** | **Event** | **Follow-up duration (1,000 person-years)** | **Incidence rate (per 1,000 person-years)** | **Model 1** | **Model 2** |
| --- | --- | --- | --- | --- | --- |
| Q1 | 2,048 | 1,953,736 | 1.05 | 1 (ref.) | 1 (ref.) |
| Q2 | 2,916 | 1,945,187 | 1.50 | 1.037 (0.979-1.099) | 1.027 (0.969-1.089) |
| Q3 | 2,595 | 1,941,393 | 1.34 | 1.027 (0.969-1.089) | 1.020 (0.962-1.081) |
| Q4 | 3,244 | 1,928,985 | 1.68 | 1.109 (1.047-1.174) | 1.084 (1.024-1.149) |

Model 1: adjusted for age, sex, smoking status, alcohol consumption, regular physical activity, low-income level, and body-mass index.

Model 2: adjusted for age, sex, smoking status, alcohol consumption, regular physical activity, low-income level, body-mass index, hypertension, diabetes, and baseline eGFR.

Abbreviation: eGFR =estimated glomerular filtration rate calculated by MDRD equation; GFR-VIM =variability independent of mean of eGFR; Q1-4 =the 1st ~ 4th quartiles of GFR-VIM.

**Table S8. Definitions of various variables used in the study.**

| **Variables** | **Definitions^*^** |
| --- | --- |
| **Smoking status** | Categorised into never smoker, ex-smoker, and current smoker, based on the answers of the questionnaire provided by the NHIS. |
| **Alcohol consumption** | Categorised into never, mild (<30 g/day) and heavy drinker (≥30 g/day), based on the answers of the questionnaire provided by the NHIS. |
| **Regular exercise** | Moderate physical activity more than 30 minutes ≥5 days/week or strenuous physical activity more than 20 minutes ≥3 days/week, based on the answers of the questionnaire provided by the NHIS. |
| **Low-income status** | Defined as if a responder belonged to the lower 10 percentile of the answers of income from the questionnaire. |

Abbreviations: NHIS =National Health Insurance Service.

*The definition is based on the questionnaire used during the health check-ups provided by the NHIS.

**Table S9. Definitions of atrial fibrillation and comorbidities used in the study.**

| **Diagnosis** | **ICD-10-CM codes** | **Diagnostic definition** |
| --- | --- | --- |
| **Atrial fibrillation** | I48.0-48.4, I48.9 | Admission or outpatient department ≥1 |
| **Hypertension** | I10-I13, I15; and minimum 1 prescription of anti-hypertensive drug (thiazide, loop diuretics, aldosterone antagonist, alpha-/beta-blocker, calcium-channel blocker, angiotensin-converting enzyme inhibitor, angiotensin II receptor blocker). | Admission ≥1 or outpatient department ≥2 |
| **Diabetes mellitus** | E11-E14; and minimum 1 prescription of anti-diabetic drugs (sulfonylureas, metformin, meglitinides, thiazolidinediones, dipeptidyl peptidase-4 inhibitors, α-glucosidase inhibitors, and insulin). | Admission ≥1 or outpatient department ≥2 |
| **Dyslipidaemia** | E78 | Admission or outpatient department ≥1 |
| **Chronic kidney disease** | None^*^ | The baseline eGFR <60 mL/min/1.73m² |

Abbreviation: eGFR =estimated glomerular filtration rate by MDRD equation; ICD-10-CM =the International Classification of Disease, Tenth Revision, Clinical Modification.

*Chronic kidney disease was defined by using the baseline eGFR instead of diagnostic codes.

**Figure S1. The cumulative incidences and risk of atrial fibrillation by the deciles of GFR-VIM.**

Abbreviation: CI =confidence interval; eGFR =estimated glomerular filtration rate calculated by MDRD equation; GFR-VIM =variability independent of mean of eGFR, HR =hazard ratio, adjusted by Model 2 (age, sex, smoking status, alcohol consumption, regular physical activity, low income level, body-mass index, hypertension, diabetes, and baseline eGFR).


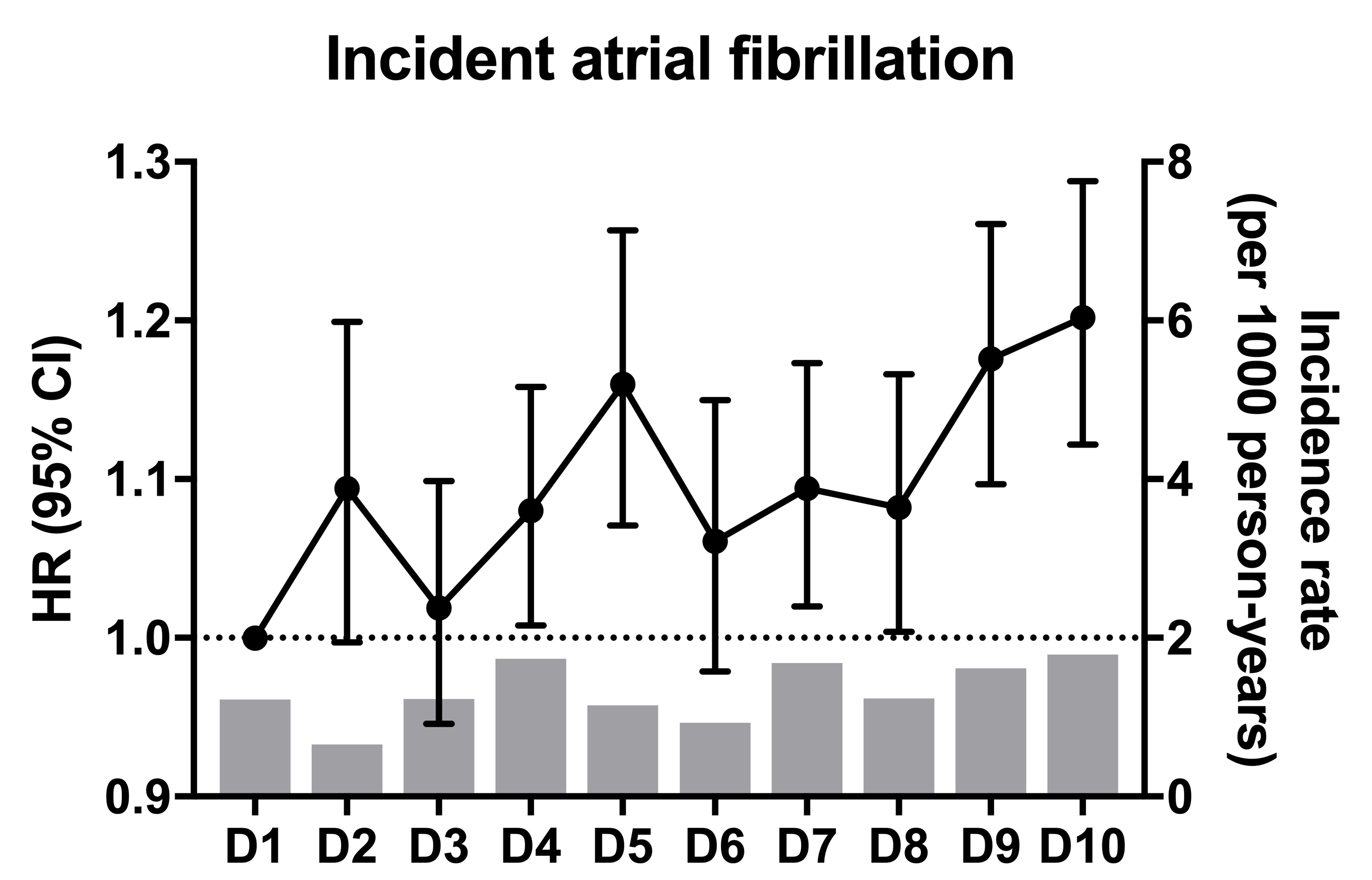


**Figure S2. The risk of incident atrial fibrillation by the variability of eGFR.**

Abbreviation: CI =confidence interval; GFR-ARV =average real variability of eGFR; GFR-CV =coefficient of variation of eGFR; GFR-SD =standard deviation of eGFR; GFR-VIM =variability independent of mean of eGFR; eGFR =estimated glomerular filtration rate calculated by MDRD equation; HR =hazard ratio, adjusted by Model 2 (age, sex, smoking status, alcohol consumption, regular physical activity, low-income level, body-mass index, hypertension, diabetes, and baseline eGFR; Q1-4 =the 1st ~ 4th quartiles of each category.


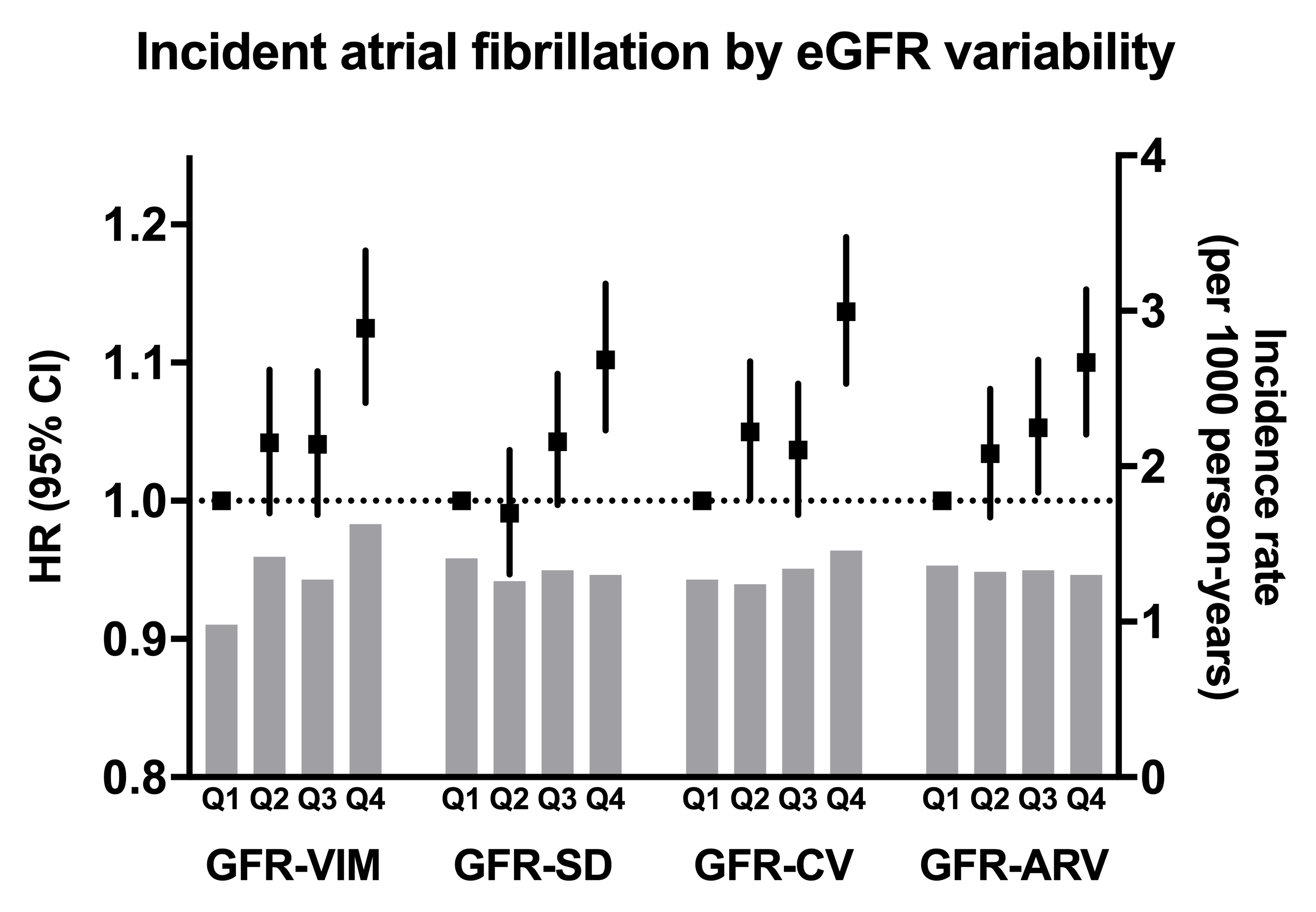


**Figure S3. The risk of incident atrial fibrillation by the variability of serum creatinine.**

Abbreviation: CI =confidence interval; Cr-ARV =average real variability of serum creatinine; Cr-CV =coefficient of variation of serum creatinine; CR-SD =standard deviation of serum creatinine; Cr-VIM =variability independent of mean of serum creatinine; eGFR =estimated glomerular filtration rate calculated by MDRD equation; HR =hazard ratio, adjusted by Model 2 (age, sex, smoking status, alcohol consumption, regular physical activity, low-income level, body-mass index, hypertension, diabetes, and baseline eGFR; Q1-4 =the 1st ~ 4th quartiles of each category.


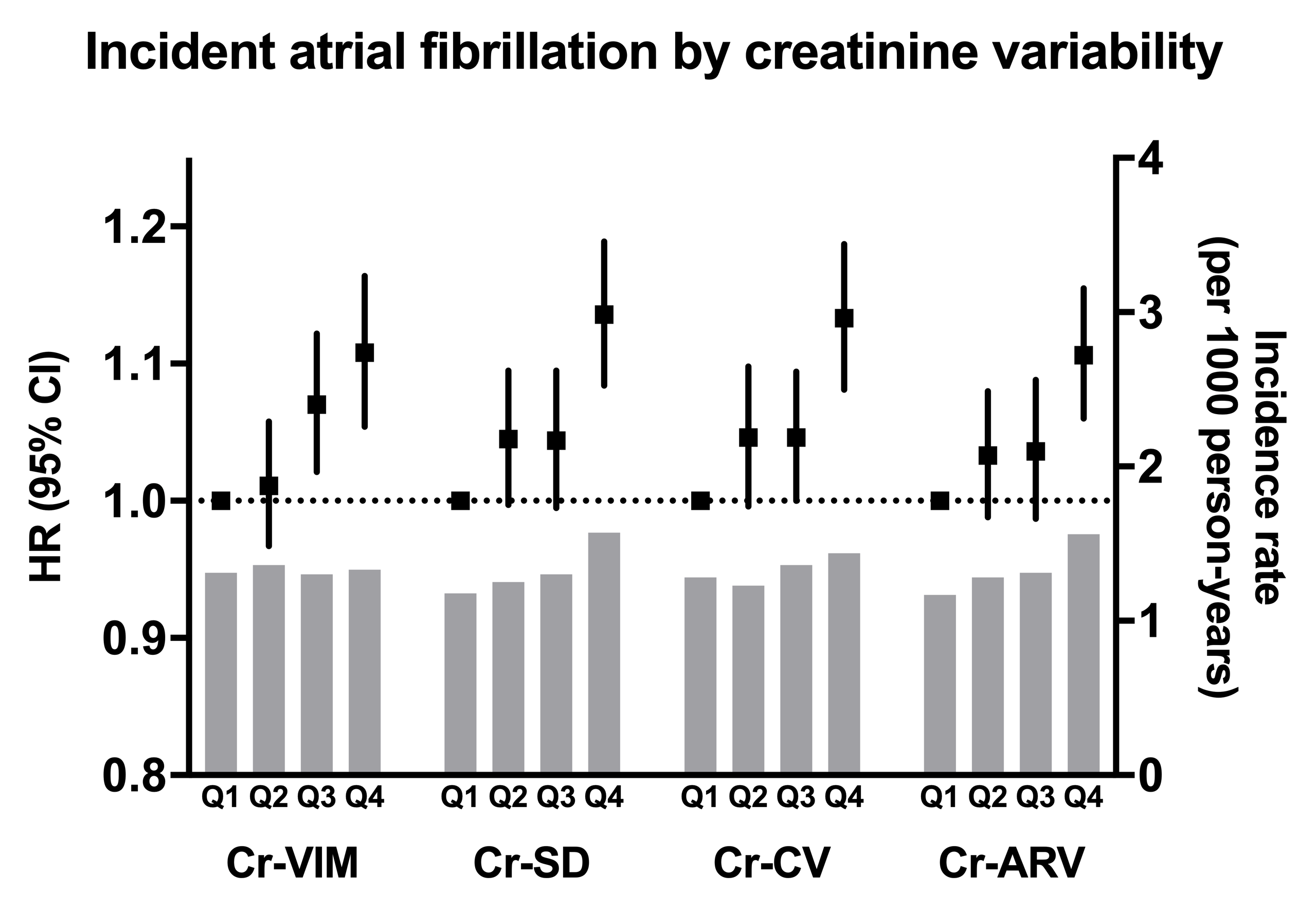


**Figure S4. The risk of incident atrial fibrillation by baseline eGFR classes**^*^**.**

Abbreviation: CI =confidence interval; eGFR =estimated glomerular filtration rate calculated by MDRD equation; GFR-VIM =variability independent of mean of eGFR; HR =hazard ratio, adjusted by Model (age, sex, smoking status, alcohol consumption, regular physical activity, low income level, body-mass index, hypertension, diabetes, and baseline eGFR); IR =incidence rate in 1,000 person-years.

*The study population was divided into three classes by the baseline eGFR (<60, 60-89, and ≥90 mL/min/1.73 m²), and the Group 1-4 for each category were re-defined by the quartiles of GFR-VIM of each class.
